# Deep learn-based computer-assisted transthoracic echocardiography: approach to the diagnosis of cardiac amyloidosis

**DOI:** 10.1007/s10554-023-02806-0

**Published:** 2023-02-10

**Authors:** Xiaofeng Zhang, Tianyi Liang, Chunxiao Su, Shiyun Qin, Jingtao Li, Decai Zeng, Yongzhi Cai, Tongtong Huang, Ji Wu

**Affiliations:** Department of Medical Ultrasonics, First Affiliated Hospital of Guangxi Medical University, No.6 Shuangyong Road, Qingxiu District, Nanning, 530021 People’s Republic of China

**Keywords:** Machine learning, Transthoracic echocardiography, Myocardial texture, Cardiac amyloidosis, Left ventricular hypertrophy

## Abstract

Myocardial amyloidosis (CA) differs from other etiological pathologies of left ventricular hypertrophy in that transthoracic echocardiography is challenging to assess the texture features based on human visual observation. There are few studies on myocardial texture based on echocardiography. Therefore, this paper proposes an adaptive machine learning method based on ultrasonic image texture features to identify CA. In this retrospective study, a total of 289 participants (50 cases of myocardial amyloidosis; Hypertrophic cardiomyopathy: 70 cases; Uremic cardiomyopathy: 92 cases; Hypertensive heart disease: 77 cases). We extracted the myocardial ultrasonic imaging features of these patients and screened the features, and four models of random forest (RF), support vector machine (SVM), logistic regression (LR) and gradient decision-making lifting tree (GBDT) were established to distinguish myocardial amyloidosis from other diseases. Finally, the diagnostic efficiency of the model was evaluated and compared with the traditional ultrasonic diagnostic methods. In the overall population, the four machine learning models we established could effectively distinguish CA from nonCA diseases, AUC (RF 0.77, SVM 0.81, LR 0.81, GBDT 0.71). The LR model had the best diagnostic efficiency with recall, F1-score, sensitivity and specificity of 0.21, 0.34, 0.21 and 1.0, respectively. Slightly better than the traditional ultrasonic diagnosis model. In further subgroup analysis, the myocardial amyloidosis group was compared one-by-one with the patients with hypertrophic cardiomyopathy, uremic cardiomyopathy, and hypertensive heart disease groups, and the same method was used for feature extraction and data modeling. The diagnostic efficiency of the model was further improved. Notably, in identifying of the CA group and HHD group, AUC values reached more than 0.92, accuracy reached more than 0.87, sensitivity equal to or greater than 0.81, specificity 0.91, and F1 score higher than 0.84. This novel method based on echocardiography combined with machine learning may have the potential to be used in the diagnosis of CA.

**Supplementary Information:**

The online version contains supplementary material available at 10.1007/s10554-023-02806-0.

## Introduction

Cardiac amyloidosis (CA) is an invasive cardiac disease characterized by the deposition of β-folded fibers within the myocardium, often with a poor prognosis. Because cardiac amyloidosis is relatively rare, the identification and evaluation of individuals are mostly limited to a few specialist centers [1]. Since amyloid deposits in the myocardium, the report should include not only wall thickness measurements but also a qualitative assessment of myocardial "texture" to aid in the diagnosis of other morphological features of cardiac amyloidosis, according to the reporting guidelines in the ASE/European Association for Cardiovascular Imaging (EACVI) guidelines [2]. Some scholars proposed that the myocardial echo of the ventricular septum and free wall of the left ventricle in CA patients was significantly enhanced with uneven distribution. The echo was presented as a ground glass image [3]. However, this subtle, non-specific and difficult to assess or quantify based on human visual observation of that myocardium.

The research on the application of artificial intelligence to myocardial diseases has gradually increased. Relevant research based on AI algorithms and radioomics mainly comes from cardiac magnetic imaging (CMR) and cardiac computed tomography (CT) [4, 5]. Although different etiology may cause differences in that change in myocardial texture on the ultrasound image. Cardiac histology studies based on echocardiography are rare because of the challenges presented by its dynamic nature [6]. This research aims at effectively identifying CA based on artificial intelligence algorithm and classification of two-dimensional gray-scale images.

## Methods

This study was approved by the Ethics Committee of the First Affiliated Hospital of Guangxi Medical University and waived the right to sign informed consent; it only involved anonymous imaging datasets; No individual patient data or human tissue samples were collected. The study was conducted in accordance with the Declaration of Helsinki (revised in 2013).

### Study population

**Fig. 1:**
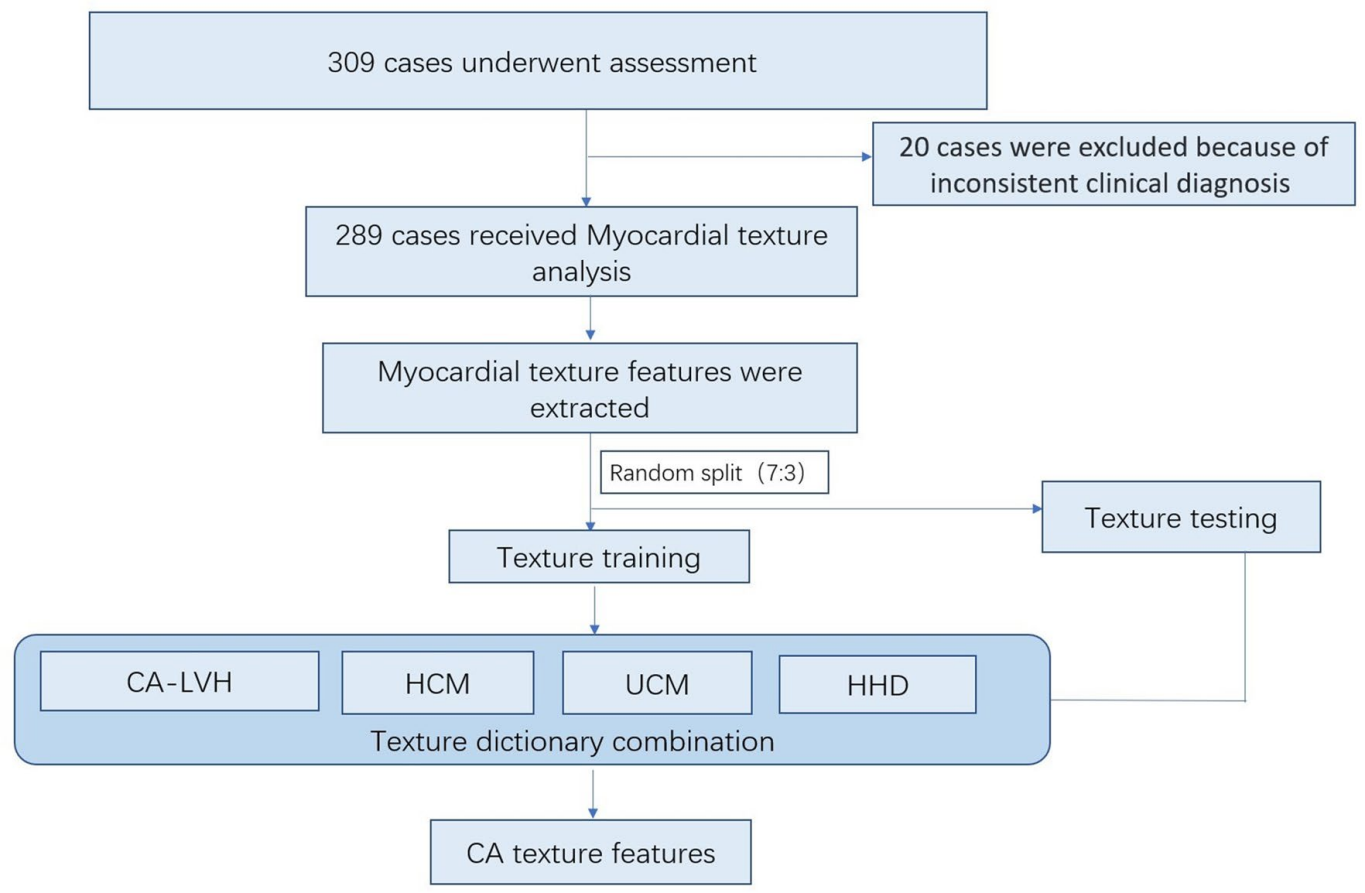
Flow chart of the enrollment procedure

A retrospective analysis of the study population was performed, and the flow chart is presented in Fig. 1. Total of 70 patients diagnosed with systemic amyloidosis in the First Affiliated Hospital of Guangxi Medical University from January 1, 2018, to April 30, 2022, were included consecutively. Left ventricular wall thickness < 11 mm at any segment of the left ventricle was excluded, and two cases with severe renal insufficiency and 17 cases without evidence of myocardial involvement were excluded. A total of 50 cases met the clinical diagnosis of CA, and were classified as the CA left ventricular hypertrophy group. Seventy-seven patients diagnosed with HHD, 70 with HCM, and 92 with UCM were included in the PACS and clinical medical record system. A total of 239 cases were in the non-CA group. All the above cases of left ventricular hypertrophy met the requirements that the left ventricular mass index (LVMI) measured by echocardiography was > 115 g/m (male) and > 95 g/m (female) [2]. The interventricular septal thickness (IVST) or the left ventricular posterior wall thickness (LVPWT) measured by ultrasound was ≥ 11 mm.

Inclusion criteria for the CA group: According to the position statement of diagnosis and treatment of cardiac amyloidosis, the diagnostic criteria are divided into invasive and non-invasive. Invasive diagnosis was used in this study: ① When the myocardial intimal biopsy showed amyloid deposition after Congo red staining; ② An extra-cardiac tissue biopsy (abdominal fat pad, rectum, kidney, bone marrow, tongue, labial glands, skin, etc.) confirms that the amyloid deposits are accompanied by cardiac amyloidosis features shown in echocardiography or by cardiac magnetic resonance (CMR) features [7].

Inclusion criteria for the non-CA group: Any one of ①, ② or ③ was met, ① Patients with HHD had a history of hypertension and no HCM or other abnormal arterial pressure load; ② In the case of HCM, other diseases causing the thickening of the left ventricular wall are excluded based on the examinations such as echocardiography or cardiac MRI showing the thickness of ventricular wall in any segment of the left ventricle ≥ 15 mm, and there is no manifestation of outflow tract obstruction [8]; ③ Patients with chronic end-stage renal disease were included in the UCM group, and the glomerular filtration rate (eGFR) < 15 mL/min/1.73 m^2^ [9].

Exclusion criteria: Cases of cardiomyopathy, severe coronary atherosclerosis, history of myocardial infarction, aortic stenosis, congenital heart disease, diabetes mellitus, athlete's heart, and insufficient image quality due to unclear etiology or multiple etiologies. Patients with UCM in combination and hypertension secondary to chronic renal failure are not excluded.

### Echocardiography

Ultrasound diagnostic instruments equipped with phased array probes were used in all cases, including GE vivid E9 (transducer M5S), Phillips IE33 (transducer S5-1), and Phillips EPIQ7c (transducer S5-1). The images were acquired and analyzed by TTE according to the echocardiography guidelines issued by the American Echocardiography Association, and the electrocardiograms were recorded synchronously. During the ultrasonic examination, the parasternal long-axis view (PLAX) of the standard parasternal left ventricle at the end-diastole was taken for analysis, the frame corresponding to the R wave peak of the electrocardiogram [2]. Ultrasound images were stored in DICOM format in the Institute's PACS, the local image archiving and communication system.

### Conventional diagnostic model

Traditional echocardiograms allow for non-invasive identification of CA based on parameters such as Relative chamber wall thickness (RWT), Electrocardiograph (ECG), pericardial effusion and Apical preservation of left ventricular long-axis strain (LS), leading to better diagnostic outcomes [10]. RWT, IVST, Apical preservation, atrial septum thickness (AST), pericardial effusion (PE), routine electrocardiograms, and tissue Doppler velocity (s′, e′, and a′) were collected in four groups of patients: CA, HCM, UCM, and HHD. The above parameters were all measured by an echocardiologist with 20 years of work experience. Convert that continuous variable into a two-class variable, and referring to an expert consensu for an optimal cutoff value, The above continuous variables were converted into two-class variables, and the optimal cutoff value was analyzed based on the optimal threshold and expert consensus [11–13]. RWT meant (IVS + PWT)/LVEDD > 0.6, IVST ≥ 12, AST > 4, Average apicalls/(average basic-ls + average mid-ls) > 1, (s′, e′, and a′) wave < 5 cm/s, and the sum of the absolute values of the highest positive and the deepest negative QRS waves of low-voltage limbs were < 0.5 mV.

### Image segmentation

**Fig. 2:**
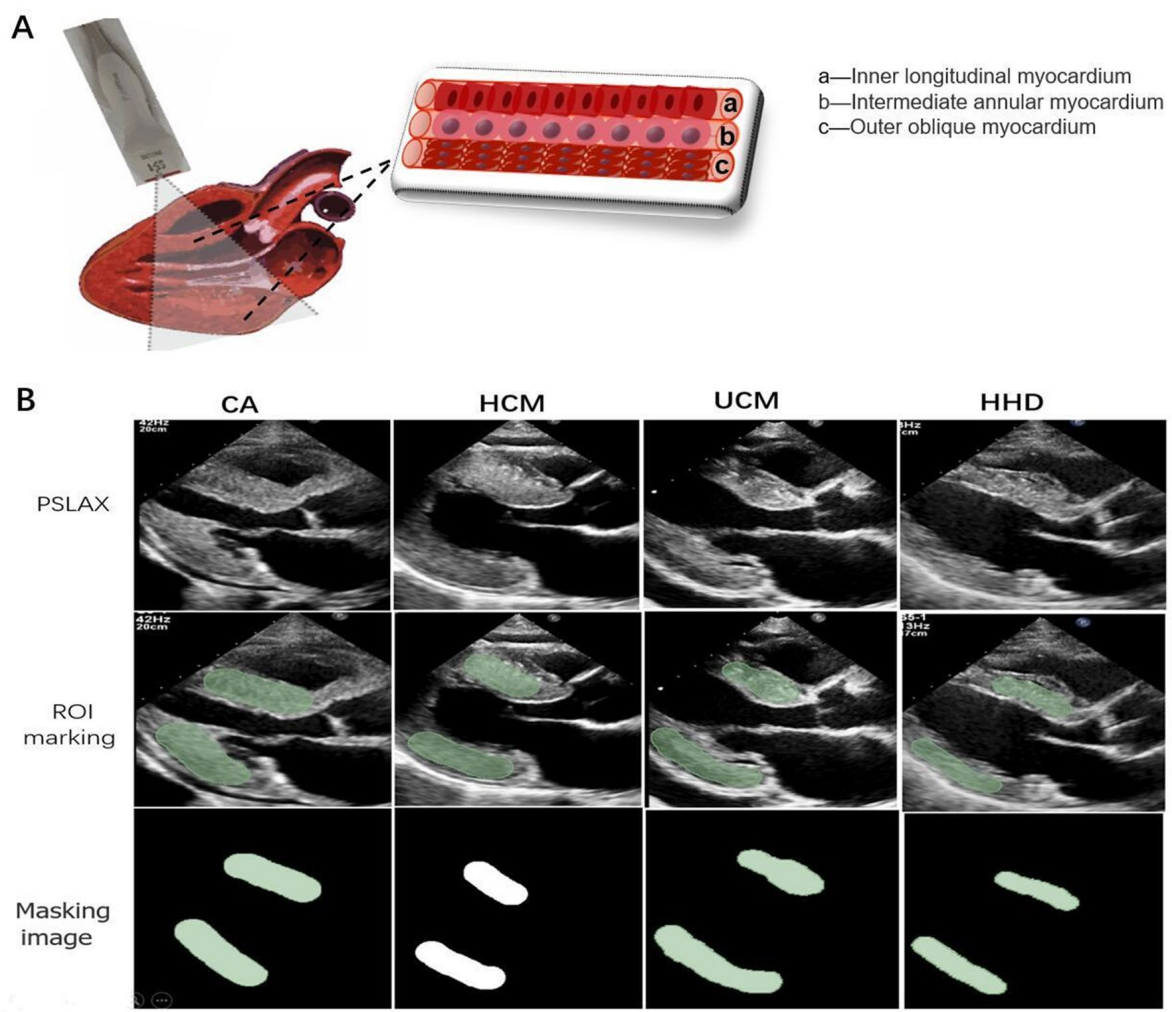
Myocardial texture feature extraction based on two-dimensional echocardiography. **A** Sketch map of PLAX section of left ventricle; **B** The region of interest (ROI) segmentation of the cardiac ultrasound image. PSLAX, parasternal long axial section of the left ventricle. ROI marking, marking of interventricular septum and posterior wall of left ventricle as region of interest. Masking image, Image binarization is performed on the extracted mask

The PLAX section selected in this study was mainly considered based on the following three points (Fig. 2A). First, myocardial texture was based on region of interest (ROI), and the larger the ROI, the better. Second, on the PLAX section, a large proportion of myocardial cells in the three layers of myocardial cells (inner longitudinal myocardium, intermediate annular myocardium and outer oblique myocardium) of the left ventricular wall ran perpendicular to the direction of the acoustic beam, with more reflected signals and strong echoes. However, the voyages of myocardium in the four-chamber or two-chamber view were mostly parallel to the direction of the acoustic beam, with relatively low reflected echoes. Third, the fan-angle energy distribution of phased array acoustic field had correlation. For the side wall located at the edge of the sound field, the reflected signals at the side lobe of the sound field was less at four-chamber or two-chamber view, and the signal attenuation was larger.

We used the 3D Slicer software (version 5.0.2; http://www.slicer.org) for region of interest (ROI) segmentation of the cardiac ultrasound image. The echocardiologist manually delineated the left ventricular posterior wall (LVPW) and the interventricular septal myocardial region as the region of interest (ROI) and manually segmented the LV myocardium using the manual offset of the epicardium and endocardium layers to avoid contamination of the epicardium or blood pool, respectively, with the image segmentation shown in Fig. 2B.

### Extraction of image omics feature

Feature extraction and image pre-processing were performed using the open-source Pyradiomics software package (version 3.0.1; https://pyradiomics.readthedocs.io/en/2.1.2/). A total of 819 radial features (18 first-order features, 73 texture features and 728 wavelet transform features) were extracted from each 2D segmentation. The feature values extracted by the training queue are normalized by the z-score; The eigenvalues of the test cohort were then normalized to z-scores using the means and standard deviations from the development cohort.

Since voxels are already isotropic in the plane, resampling is not required before feature extraction. On the other hand, gray-level overall image standardization was performed to ensure comparability of images obtained with different scanners and different Settings, with results ranging from 0 to 600. Wavelet decompositions represent an alternative approach to remove low signal areas from the images (i.e., image smoothing and edge detection). Using high- and low-pass filter combinations, the original image is decomposed in distinct components, expanding the original signal. As the best practice for medical image analysis is not established, these alternative filtering approaches have been both included in the investigation [14].

For the feature classes, 2D shape was discarded and the first order, Gray-level Co-occurrence Matrix (GLCM), Gray-level size zone matrix (GLZM), gray-level run-length matrix (GLRLM), neighbourhood gray-level dependency matrix (GLDM), and neighbourhood gray-level difference matrix (NGTDM) were extracted. Since the 2D image was based on as much myocardial texture information as possible of the delineation, the 2D shape feature is not specific. For the remaining classes, all available features were calculated except for the GLCM and the mean values, which was a recommendation of the PyRadiomics developer due to the known redundancy of the other GLCM parameters.

Formulas and definitions of the extracted features can be found on the official documentation (https://pyradiomics.readthedocs.io/en/latest/features.html).

### Data analysis and feature selection

Two sonographers (C.S., 5 years of experience in cardiac imaging; S.Q., 20 years of experience in cardiac imaging) participated in ROI segmentation, respectively. First, all ROI was segmented by Doctor 1. In order to evaluate the feature repeatability within the observer, Doctor 1 randomly selected 30 patients to perform ROI segmentation after a 4 week washout period. To evaluate the feature repeatability between observers, Doctor 2 independently segmented the ROI of the same 30 patients and evaluated the reliability of the features by calculating the intra-class and inter-observer correlation coefficient (ICC).

**Fig. 3:**
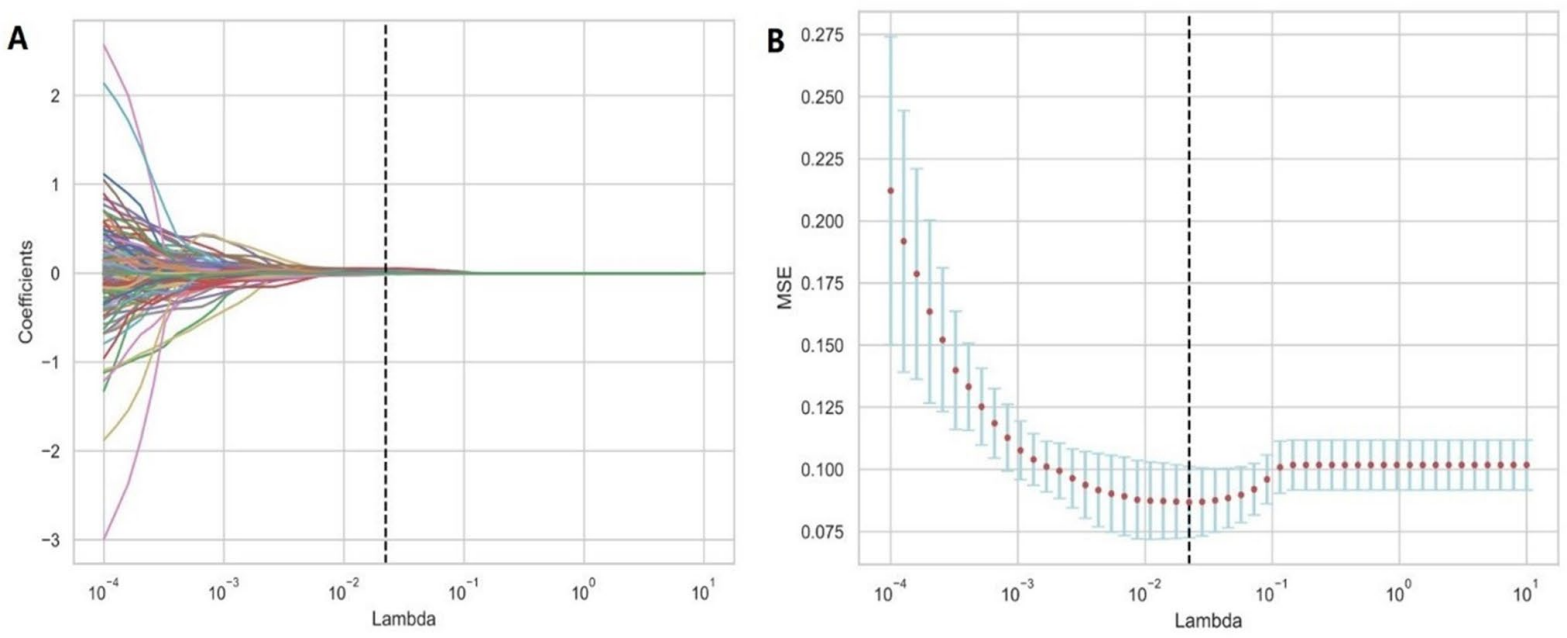
Using the least absolute shrinkage and selection operator (Lasso) logistic regression model to select ultrasound features associated with myocardial amyloidosis. **A** The coefficient convergence graph for the feature selection process. The ordinate represents the respective coefficient of the feature in the model, and the abscissa is log(λ); **B** The ordinate is the binomial deviation, and the abscissa is log(λ). The binomial deviation of the model is minimized by adjusting different parameters of λ so as to screen out indicators with higher diagnostic value

In order to identify the robust imageological features, we conducted a three-step screening. First, the features with high intra-observer and inter-observer stability (correlation coefficient > 0.80) were retained. Then, the image features with significant differences between CA and nonCA were screened out using the student-t test (*P* < 0.05). Finally, the final features are screened out using the minimum absolute contraction and selection algorithm (Lasso regression) to construct the imageological feature model (Fig. 3).

Texture features are feature sets derived from the image pixel distribution matrix, which has been proved to be the most effective feature set for distinguishing pathological types in many studies. Therefore, five types of texture correlation matrices commonly used in previous studies were selected in this study, including Gray-level Co-occurrence Matrix (GLCM), Gray-level size zone matrix (GLZM), gray-level run-length matrix (GLRLM), neighbourhood gray-level dependency matrix (GLDM), and neighbourhood gray-level difference matrix (NGTDM) [15, 16].

Wavelet transform can obtain multi-frequency domain and multi-scale image information. For the clinical problem that is difficult to describe with simple visual features of tumor images, the high-dimensional abstract feature of wavelet features may play a different role in capturing the clinical information that is not easily perceived by vision [17].

### Establishment of classification model

In this study, four classical classification learning methods, including random forest (RF), support vector machine (SVM), logical regression (LR), and gradient decision tree (GBDT), were used to model the data.

RF belongs to a supervised learning algorithm, a bagged ensemble learning algorithm based on a decision tree-based learner. A tree is constructed by random selection of training data sets, and features are randomly selected. Finally, the results of all the decision trees are averaged or voted to obtain the results. SVM is the most concerned algorithm in machine learning. It has a good effect on linear and nonlinear classification problems and performs well in various practical problems. It is widely used in handwritten numeral recognition and face recognition [18]. LR logistic regression is a classification algorithm aiming to find the relationship between features and the probability of specific results. The main advantage of this method is that the probability is limited between 0 and 1. GBDT is a boosting-type ensemble learning algorithm. A strong learner is gradually constructed by combining the ability of weak learners to constantly predict the difficult-to-learn samples. GBDT has the same strong generalization ability as SVM and has been widely used in search ranking and other fields in recent years [19].

### Evaluation methodology

The data were randomly divided into a 7:3 ratio of the training set to test set (202 cases in the training set and 87 cases in the test set) for learning, and the accuracy, sensitivity, and specificity of the myocardial prediction model for myocardial amyloidosis and the AUC of ROC curve were verified on the test set.

### Statistical analysis

Continuous variables were described as mean ± SD, and enumeration data were compared among groups using χ^2^ test or Fisher exact probability method. Bonferroni method was used to correct the significance level for pairwise comparison. *P* < 0.05 indicated that the difference was statistically significant. The area under the curve, AUC), accuracy, and specificity of the receiver operating characteristic curve were used to evaluate the performance of each machine learning model in the classification task. Feature extraction, feature dimensionality reduction, and modeling are performed on open source tool python (version: 3. 9. 7, https://www.python.org/) software. SPSS 23.0 statistical software was used for the correlation test.

## Results

### Patient characteristics and echocardiography

The patient demographic characteristics and routine echocardiogram parameters are presented in Table 1. Compared with the nonCA group, the LVPW of the CA group was the thickest, and the E/A and LVEF were the lowest. The HCM group showed maximum IVS thickness, LVMI, and minimum LVEDD, E/e′. E/e′ in the UCM group was higher than in other groups, suggesting that diastolic function was significantly decreased. The values of IVS, LVPW and LVMI in the HHD group were the smallest, and the *P* values in the one-way analysis of variance were all < 0.05. Of course, atrial fibrillation (AF) cases were excluded from the E/A and E/e′ analyses.

**Table 1:** Patient characteristics and routine echocardiography

Characteristics	CA	nonCA			One-way ANOVA	
		HCM	UCM	HHD	F	P
No. of cases	50	70	92	77		
Age (year)	58.8 ± 6.7	49.9 ± 13.0*	52.2 ± 9.4*	54.9 ± 8.1	9.205	< 0.001
Female, n(%)	13(26)	21(30)	33(35.9)	21(27.3)	X^2^ = 2.117	0.587
BSA, m2	1.70 ± 0.11	1.61 ± 0.13*	1.63 ± 0.10	1.69 ± 008	11.044	< 0.001
Low-ECG, n(%)	13, (26.0)	0, (0)	15, (16.3)	0, (0)		< 0.001
Echocardiography						
IVS thickness (mm)	15.3 ± 1.4	16.5 ± 0.7*	14.6 ± 0.8*	12.7 ± 07*	226.998	< 0.001
LVPW thickness (mm)	14.5 ± 1.4	11.2 ± 0.9	13.9 ± 0.7*	12.0 ± 06*	114.008	< 0.001
** L**VMI(g/m')	169.6 ± 24.8	156.4 ± 22.7*	176.3 ± 19.9	135.7 ± 22.3*	65.166	< 0.001
LVEDD (mm)	46.4 ± 4.6	45.8 ± 2.7	48.3 ± 2.5*	47.9 ± 3.6	10.202	< 0.001
LVEF	0.57 ± 0.08	0.65 ± 0.06*	0.59 ± 0.07	066 ± 005*	31.123	< 0.001
E/A ratio	0.65 ± 0.10	0.86 ± 0.33	1.00 ± 0.51*	082 ± 019	10.876	< 0.001
E/e′ ratio	12.0 ± 2.3	9.1 ± 1.6*	14.6 ± 2.1*	12.3 ± 1.4*	219.127	< 0.001
RWT	0.60 ± 0.04	0.52 ± 0.04*	0.62 ± 0.09	06 ± 0.05	38.236	< 0.001
IAS	2.3 ± 0.9	1.5 ± 0.2*	1.7 ± 0.2*	1.6 ± 02*	40.387	< 0.001
"5-5-5"sign, n(%)	9, (18.0)	0, (0)	30, (32.6)	0. (0)	X^2^ = 52.455	< 0.001
Apical-sparing, n(%)	14, (28.0)	0, (0)	0, (0)	0, (0)		< 0.001
PE, n(%)	37, (74.0)	2, (2.9)	34, (64.0)	4, (5.2)	x^2^ = 100.766	< 0.001

*CA* cardiac amyloidosis, *nonCA* non cardiac amyloidosis, *HCM* hypertrophic cardiomyopathy, *UCM* uremic cardiomyopathy, *HHD* hypertensive heart disease, *BSA* body surface area, *IVS* interventricular septum thickness, *LVPW* left ventricular posterior wall, *LVMI* left ventricular mass index, *LVEDD* left ventricular end-systolic diameter, *LVEF* left ventricular ejection fraction, *E* early peak velocity of trans-mitral LV filling, *e′* diastolic early peak tissue velocity at the mitral annulus olic volume, *A* late peak velocity of trans-mitral LV filling, *"5-5-5"sig* all tissue Doppler imaging velocities < 5 cm/sec, *Apical-sparing* average apical LS/(average combined mid + base LS) > 1, *PE* pericardial effusion, *low-ECG* Low/decreased QRS voltage to degree of LV thickness

Values are mean ± SD. Compared with CA group

*P < 0.001

In this study, logical regression analysis was used to identify CA and nonCA based on conventional echocardiography parameters such as RWT and left ventricular strain together with ECG results, which initially indicated a good identification effect. An overall accuracy of 0.80 was obtained with recall, F1-score, sensitivity, specificity, and AUC of 0.29, 0.45, 0.29, 1.0, and 0.76, respectively.

### Feature extraction and selection

**Fig. 4:**
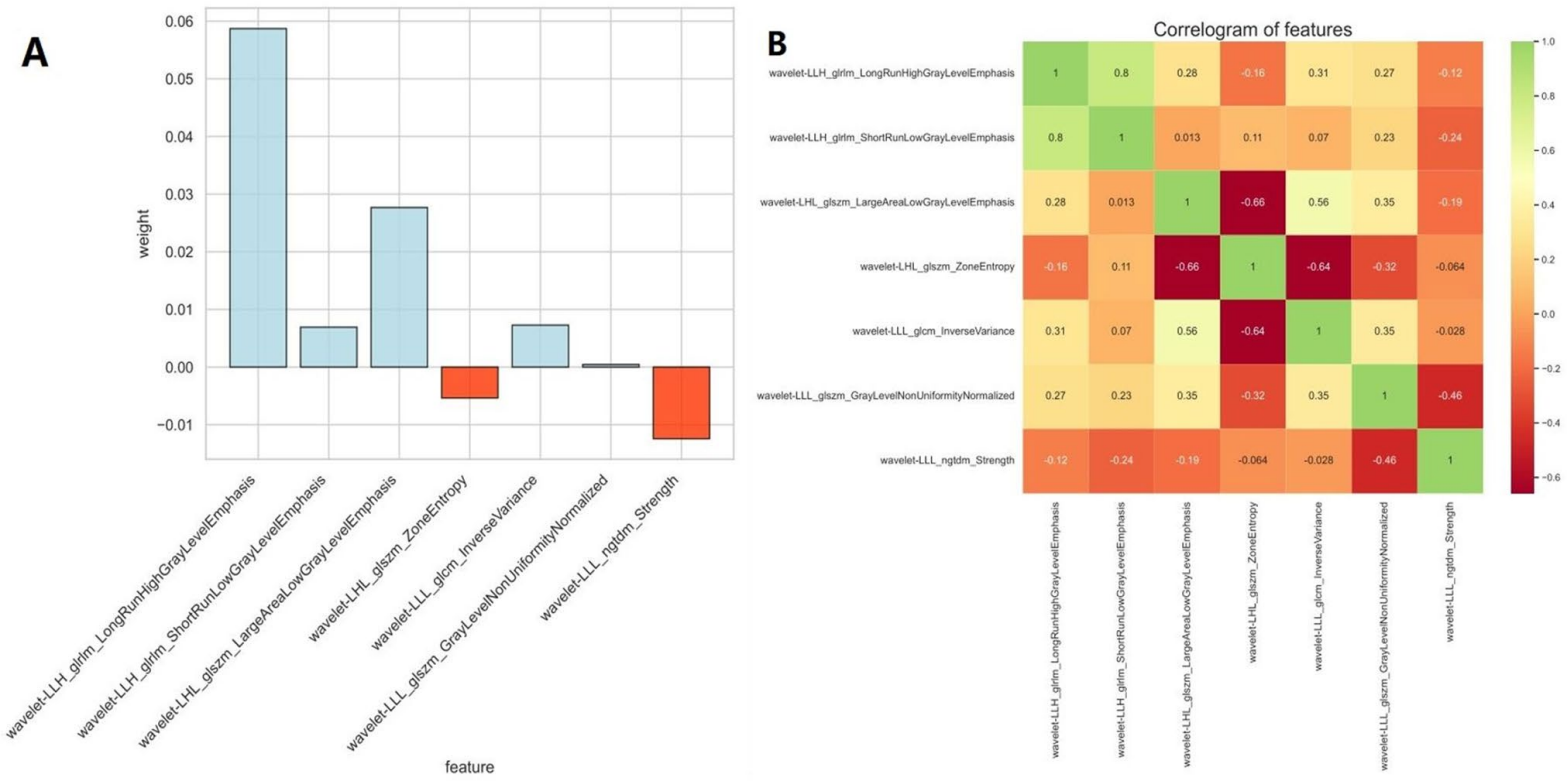
Screening results of myocardial texture characteristics in CA and nonCA groups. **A** Weight coefficients of each myocardial texture feature. **B** Correlogram illustrating independence between each texture feature. Red squares represent negative correlations, while green squares represent positive correlations. The magnitude of the correlation between features is indicated by the color gradient

**Fig. 5:**
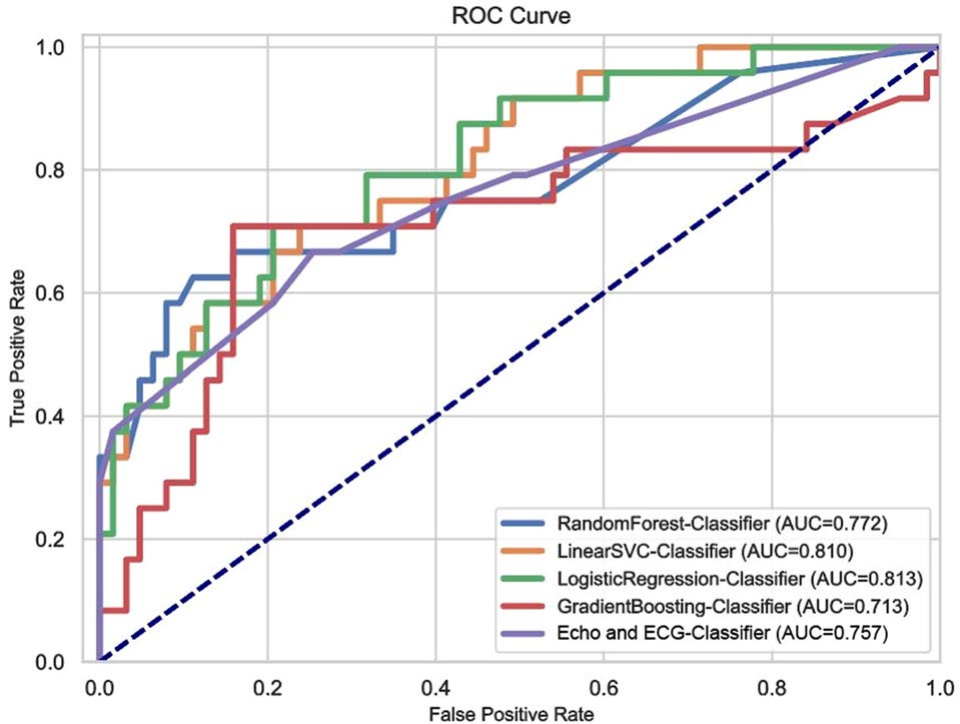
ROC curves of the models for distinguishing CA group and nonCA group were constructed based on four algorithms. **A** Receiver operating characteristic curve of the prediction model based on random forest. **B** Receiver operation characteristic curve of the prediction model based on SVM. **C** Receiver operation characteristic curve based on logistic regression. **D** Receiver operation characteristic curve of the prediction model based on GBDT

Seven radiographic features were finally screened in the overall population to establish the model: Wavelet-LLH-GLLM-LonggrunhighGrieLevelEmphasis, Wavelet-LLH-GLLM-ShortrunLowGrieLevelEmphasis. Wavelet-LHL-GLSZM-LargeAreaLowGrayLevelEmphasis, Wavelet-LHL-GLSZM-ZoneEntropy, Wavelet-LLL-GLCM-InverseVariance, Wavelet-LLL-GLSZM-GrayLevelNonUniformityNormalized, Wavelet-LLL-NGTDM-Strength. Figure 4 shows the coefficients and correlation of these seven features. We established four machine learning models, including RF, SVM, LR and GBDT, all of which had good performance: AUC values were 0.77, 0.81, 0.81 and 0.71 (Fig. 5), and the accuracy rate was 0.88, LR had the best diagnostic efficiency with recall, F1-score, sensitivity and specificity of 0.21, 0.34, 0.21 and 1.0, respectively (Table 2).

**Table 2:** Prediction performance of each classifier in CA and nonCA datasets

Parameters	AUC	Accuracy	Sensitivity	Specificity	F1 score
RF	0.77	0.77	0.16	1.0	0.28
SVM	0.81	0.75	0.08	1.0	0.15
LR	0.81	0.78	0.21	1.0	0.34
GBDT	0.71	0.75	0.25	0.94	0.35

*RF* random forests, *SVM* support vector machine, *LR* logistic regression, *GBDT* gradient boosting decision tree. *AUC* area under the curve

### Comparative analysis of subgroup model

*Comparison of myocardial texture feature between CA and HCM groups,* Through the same feature screening method above, eight optimal features for identifying CA and HCM were screened, including two first-order texture features including GLRLM and NGTDM matrix features, six texture features after wavelet transform, and the supplementary materials for revealing the coefficients and correlation between the texture features (Fig. S1 A, B).

The selected texture features were modeled by the four classification algorithms mentioned above. The AUC values for model evaluation reached above 0.82, and the accuracy reached above 0.72. The sensitivity was greater than or equal to 0.53, specificity was higher than 0.76, and F1 score was higher than 0.62. See Table 3 for the ROC curves of the four models and the supplementary materials (Fig. S2).

**Table 3:** Prediction performance of each classifier in CA and HCM datasets

Parameters	AUC	Accuracy	Sensitivity	Specificity	F1 score
RF	0.88	0.75	0.6	0.86	0.67
SVM	0.82	0.75	0.73	0.76	0.71
LR	0.85	0.75	0.73	0.76	0.71
GBDT	0.82	0.72	0.53	0.86	0.62

*RF* random forests, *SVM* support vector machine, *LR* logistic regression, *GBDT* gradient boosting decision tree. *AUC* area under the curve

*Comparison of myocardial texture features between CA and UCM groups,* Through the same feature screening method mentioned above, a total of 20 features, one first-order GLDM matrix feature and 19 texture features after wavelet transform are screened from the optimal subsets of identification CA and UCM, and the texture feature coefficients and correlation are shown in the supplementary materials (Fig. S3 A, B).

The selected texture features were modeled by four classification algorithms. The AUC values evaluated by the four models were RF:0.79, SVM:0.84, LR:0.82, and GBDT:0.75 (Fig. S4), with an accuracy rate of more than 0.67, and the sensitivity of 0.59 or higher, specificity of 0.73 and F1 score of 0.59, respectively, as shown in Table 4. The ROC curves of the four models are shown in the supplementary materials (Fig. S4).

**Table 4:** Prediction performance of each classifier in CA and UCM datasets

Parameters	AUC	Accuracy	Sensitivity	Specificity	F1 score
RF	0.79	0.74	0.65	0.81	0.67
SVM	0.84	0.81	0.76	0.85	0.76
LR	0.82	0.77	0.71	0.81	0.71
GBDT	0.75	0.67	0.59	0.73	0.59

*RF* random forests, *SVM* support vector machine, *LR* logistic regression, *GBDT* gradient boosting decision tree. *AUC* area under the curve

*Comparison of myocardial texture feature between CA and HHD groups,* Through that same feature screen method, a total of 23 features to identify the optimal subsets of the CA and the HHD are screened, and the five first-order texture features comprise a first-order quartile deviation and total energy and three original GLSZM matrix features; For the texture features after the 19 wavelet transforms, the texture feature coefficients and the correlation between the features, please refer to the supplementary materials (Fig. S5 A, B).

The selected texture features were modeled by four classification algorithms, respectively. The AUC value of model evaluation was above 0.92, the accuracy rate was above 0.87, the sensitivity was greater than or equal to 0.81, the specificity was 0.91, and the F1 score was above 0.84. The ROC curves of the four models and the supplementary materials (Fig. S6).

## Discussion

In this study, we distinguished CA from non-CA through the ultrasonic imaging omics and machine learning model, which can provide an excellent and non-invasive diagnostic tool for clinical practice. The machine learning model was slightly better than conventional echocardiography combined with electrocardiography for multi-parameter identification, such as RWT and left ventricular strain, AUC (0.81 vs 0.76).

The pathological condition of abnormal morphology and function caused by the deposition of amyloid fibers in the cardiac interstitium is called cardiac amyloidosis [20, 21]. Larsen et al. [22] reported differences in the histopathological findings of ATTR amyloidosis and AL amyloidosis. They reported that the extent and distribution of cardiac amyloid deposits correlated with amyloid type, indicating fundamental differences in the pathobiology of deposition. Imaging revealing this pathological difference can be challenging, especially for a primary echocardiologist, requiring a comprehensive analysis of multiple imaging data and clinical information. The echocardiographic findings that suggested infiltrative disease include normal to small LV cavity size, biatrial enlargement and dysfunction, left atrial and left atrial appendage stasis and thrombi, thickened valves, right ventricular and interatrial septal thickening, pericardial effusion and a restrictive transmitral Doppler filling pattern [23, 24]. In particular, “apical sparing”, a key feature distinguishing patients with CA from those with other types of left ventricular hypertrophy, and reflecting myocardial involvement in the early stage of the disease, was calculated as per the following formula: average apical LS/(average basal-LS + average mid-LS) [11, 25]. Although apical sparing performed well with specificity, it only performed well in the mid-term of the disease and the ultrasonic strain parameters were not specific in the early and late CA.

CMR is currently the first-line diagnostic technique for CA, but amyloidosis is often accompanied by renal abnormalities, which limits the use of gadolinium-containing contrast agents, and CMR cannot be performed in most primary hospitals [26]; Endomyocardial biopsy is the gold standard for the diagnosis of CA, but it is invasive, difficult to operate, and has high examination risk and complication probability [27]. TTE is more convenient and economical than CMR and cardiac CT and is more commonly used in community hospitals. In previous studies, two-dimensional echocardiography showed other features such as papillary muscle thickening, valvular thickening, better visualization of right ventricular wall thickening, and the characteristic "granular flash" appearance of the thickened ventricular wall [28].

Previous researchers have reported the feasibility and usefulness of myocardial texture analysis based on CMR, cardiac CT and echocardiography in several myocardial abnormalities such as myocardial infarction, myocarditis, myocardial fibrosis, HCM and etiological identification of left ventricular hypertrophy [5, 29, 30]. Most applications of artificial intelligence in echocardiography have focused on improving image acquisition automation and tedious, repetitive tasks. Few studies have shown that artificial intelligence can provide clinically important insights from the subtle and non-specific data represented by myocardial texture [31]. Our research group applies the static myocardial texture of ultrasound B-mode images to explain the subtle changes in myocardial texture in different diseases based on the algorithm of artificial intelligence and converts digital medical images into high-dimensional data that can be mined. The idea is that biomedical images contain information reflecting potential pathophysiology, and the relationship can be revealed through quantitative image analysis [32].

The present study offers an attractive alternative subjective interpretation of conventional ultrasound, as assessed by the clinical B-mode ultrasound. Myocardial texture features provide detailed quantification of the texture changes, which improves the nature of the current subjective interpretation of ultrasound images [30]. Although the four models a good performance, the sensitivity and F1 score of these classification methods for identifying the CA group and nonCA group were relatively low, which might be related to the small number of CA groups in this sample size. In further subgroup analysis, the CA group was compared with the HCM group, the UCM group, and the HHD group, and the same method was used for feature extraction and data modeling. The model diagnosis efficiency was further improved. In particular, 23 myocardial texture features in the identification CA group and the HHD group were evaluated by four classification learning models, and the AUC value was above 0.92, the accuracy rate was above 0.87, the sensitivity was greater than or equal to 0.81, the specificity was 0.91, and the F1 score was above 0.84. As mentioned by Lambin et al. [33], a good image omics feature should work well with different classifiers.

### Study limitations

The current study has several limitations that need further consideration. First, the number of cases of CA in this study was relatively small, and the sensitivity of the model constructed for the identification of the CA group and nonCA group might be low. Therefore, pairwise comparison evaluation was adopted for further subgroup analysis. This study was a single-center retrospective study using ultrasound images obtained in only one laboratory. The nonCA group was only included in the HCM, UCM, and HHD groups that commonly cause left ventricular hypertrophy but not included in the relatively rare cardiomyopathy that causes left ventricular hypertrophy, such as Fabry's disease and aortic stenosis, which may have a certain selectivity bias. The seven parameters included in traditional diagnostic models did not include evidence of N-terminal pro-brain natriuretic peptide, monoclonal immunoglobulins or free light chains in blood or urine, as these tests were not performed in all cases. In addition, this study had many variables, such as chest wall thickness, the distance between the probe and heart, and exploration angle, which might affect image quality. In addition, the time and financial cost of this in-depth learning diagnostic model are significantly lower than those of the traditional echocardiography diagnostic model, but detailed statistics have not been performed in this study.

## Conclusion

In this study, computer-aided transthoracic echocardiography based on depth learning proposed a new non-invasive diagnostic method for myocardial amyloidosis, which diagnostic efficiency was slightly superior to that of traditional multi-parameter combination. The artificial intelligence-based myocardial texture analysis using conventional two-dimensional transthoracic echocardiography could effectively distinguish CA left ventricular thickening from nonCA left ventricular thickening.

## Supplementary Information

Below is the link to the electronic supplementary material.

Supplementary file1 (DOCX 1176 kb)

## Abbreviations

CA: Myocardial amyloidosis
LVH: Left ventricular hypertrophy
TTE: Transthoracic echocardiography
HCM: Hypertrophic cardiomyopathy
UCM: Uremic cardiomyopathy
HHD: Hypertensive heart disease
CMR: Cardiac magnetic imaging
CT: Cardiac computed tomography
AUC: Area under the curve
ROC: Receiver operating characteristic
LVMI: Left ventricular mass index
IVST: Interventricular septal thickness
LVPWT: Left ventricular posterior wall thickness
ICC: Inter-observer correlation coefficient
ROI: Region of interest
GLCM: Gray-level co-occurrence matrix
GLZM: Gray-level size zone matrix
GLRLM: Gray-level run-length matrix
GLDM: Neighborhood gray-level dependency matrix
NGTDM: Neighborhood gray-level difference matrix
RF: Random forest
SVM: Support vector machine
LR: Logical regression
GBDT: Gradient decision tree
