# Supplementary material for: Deep learn-based computer-assisted transthoracic echocardiography: approach to the diagnosis of cardiac amyloidosis
Source: Int J Cardiovasc Imaging. 2023 Feb 10;39(5):955–65. doi: 10.1007/s10554-023-02806-0 (PMC10159959; doi:10.1007/s10554-023-02806-0)
Supplement: Supplementary file 1 — Supplementary file1 (DOCX 1176 kb) [file 10554_2023_2806_MOESM1_ESM.docx]

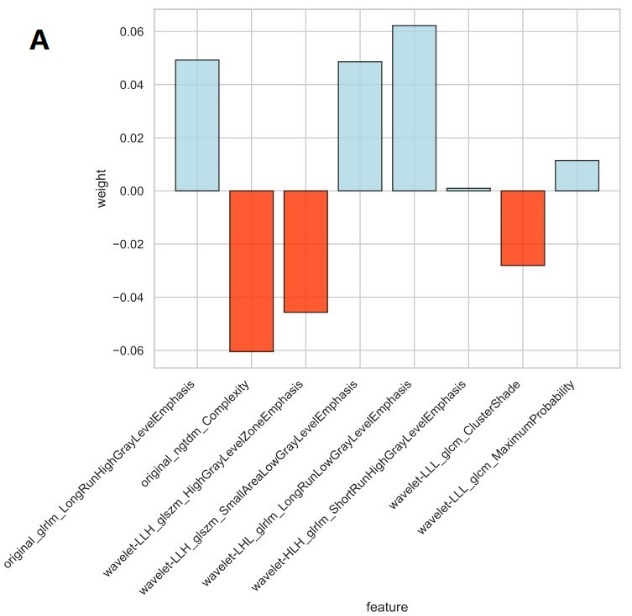

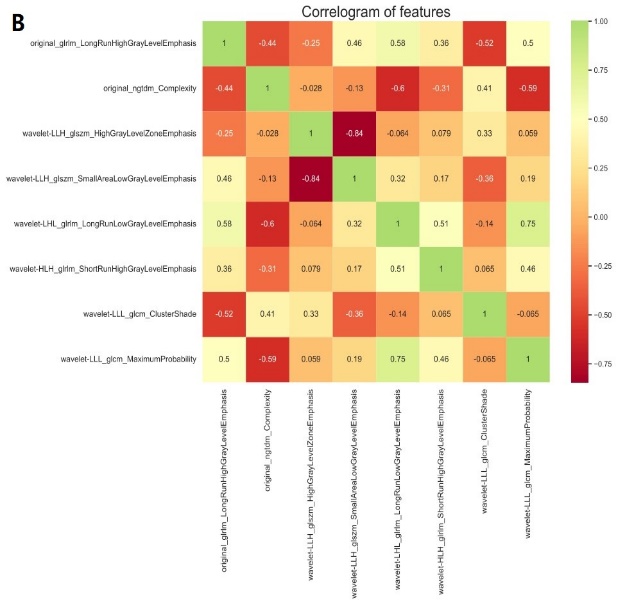


Figure S1 Screening results of myocardial texture characteristics in CA-LVH and HCM groups. (A) Weight coefficients of each myocardial texture feature. (B) Correlogram illustrating independence between each texture feature. Red squares represent negative correlations, while green squares represent positive correlations. The magnitude of the correlation between features is indicated by the color gradient.


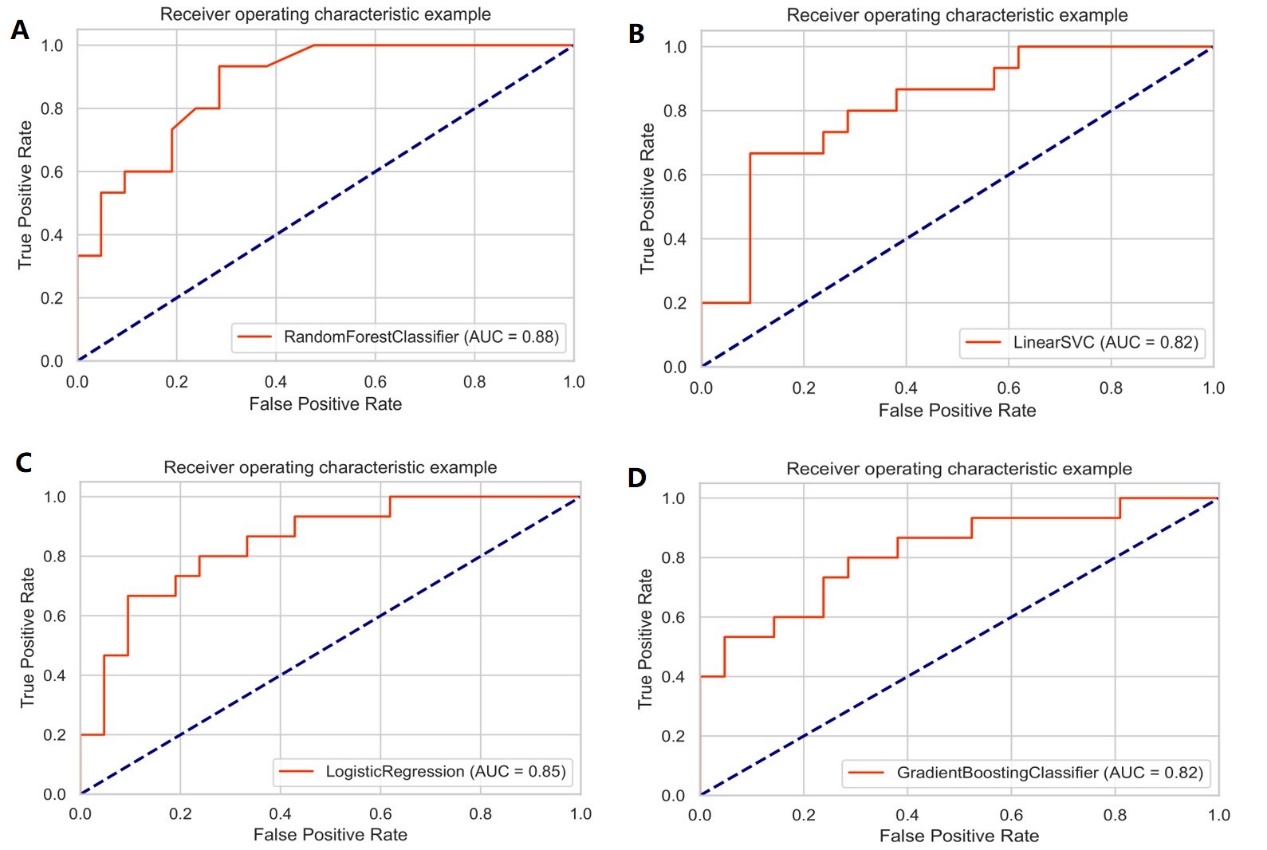


Figure S2. ROC curves of the ca-LVH group and HCM group models were constructed based on four algorithms. (A) Receiver operating characteristic curve of the prediction model based on random forest. (B) Receiver operation characteristic curve of the prediction model based on SVM. (C) Receiver operation characteristic curve based on logistic regression. (D) Receiver operation characteristic curve of the prediction model based on GBDT.


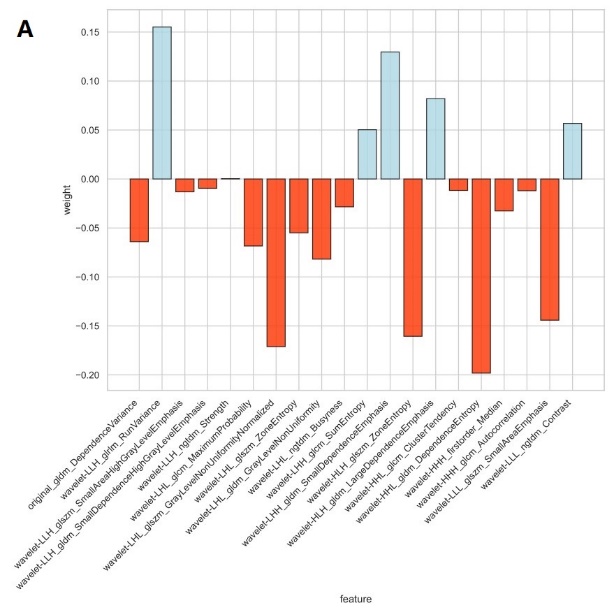

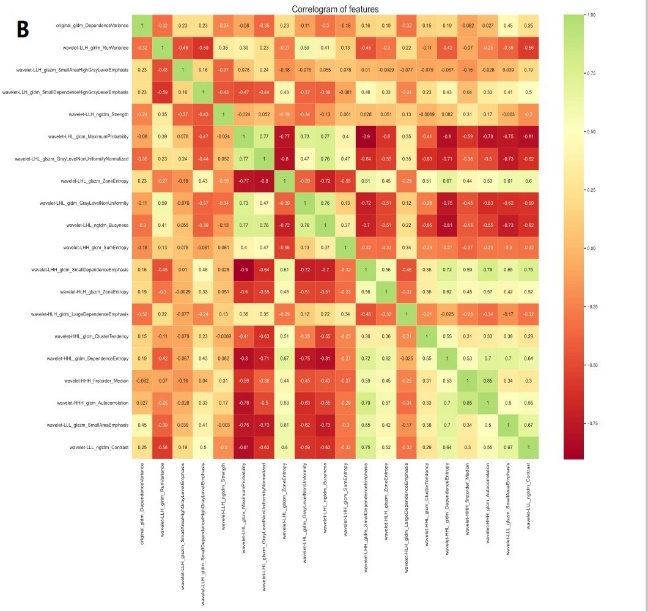


Figure S3 Screening results of myocardial texture characteristics in CA-LVH and UCM groups. (A) Weight coefficients of each myocardial texture feature. (B) Correlogram illustrating independence between each texture feature. Red squares represent negative correlations, while green squares represent positive correlations. The magnitude of the correlation between features is indicated by the color gradient.


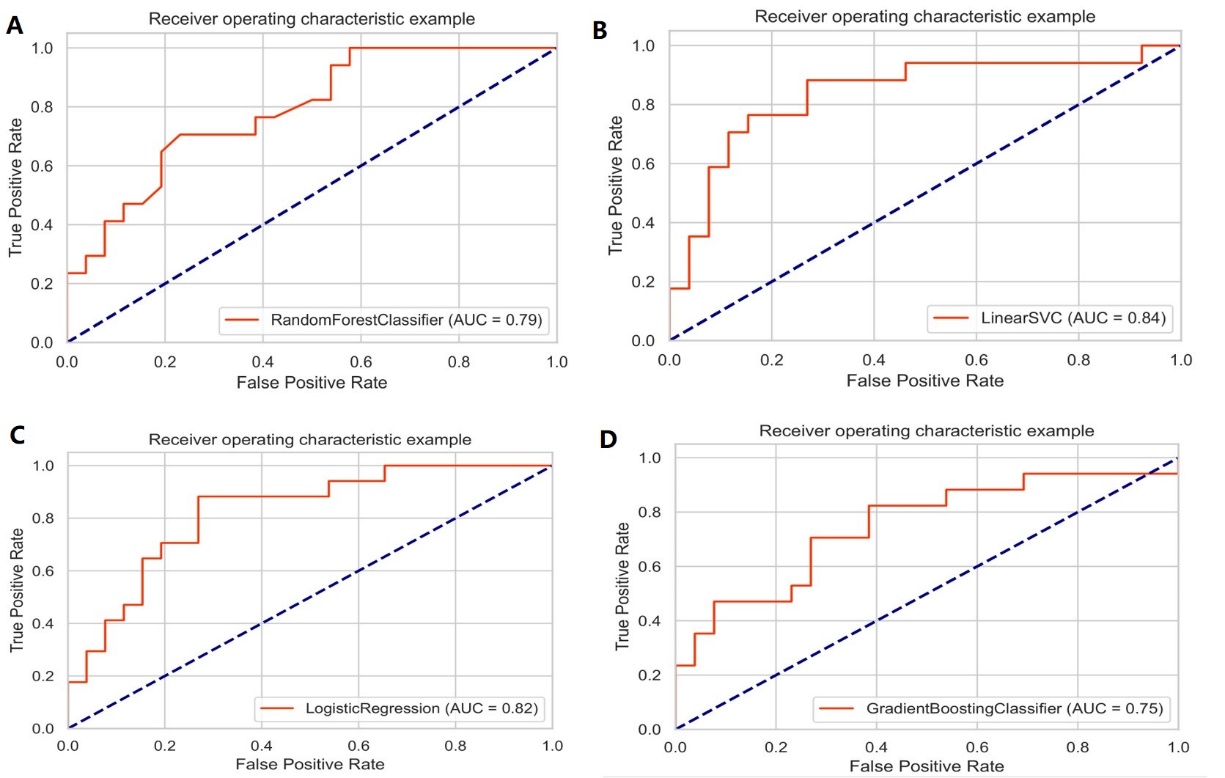


Figure S4. ROC curves of the ca-LVH group and UCM group models were constructed based on four algorithms. (A) Receiver operating characteristic curve of the prediction model based on random forest. (B) Receiver operation characteristic curve of the prediction model based on SVM. (C) Receiver operation characteristic curve based on logistic regression. (D) Receiver operation characteristic curve of the prediction model based on GBDT.


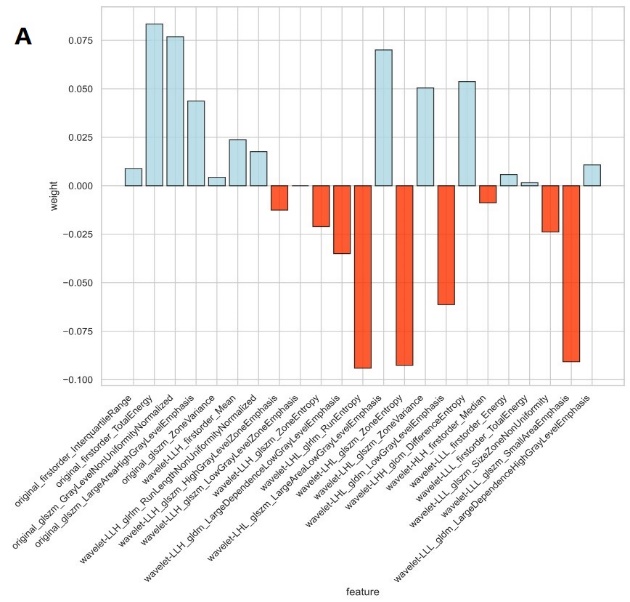

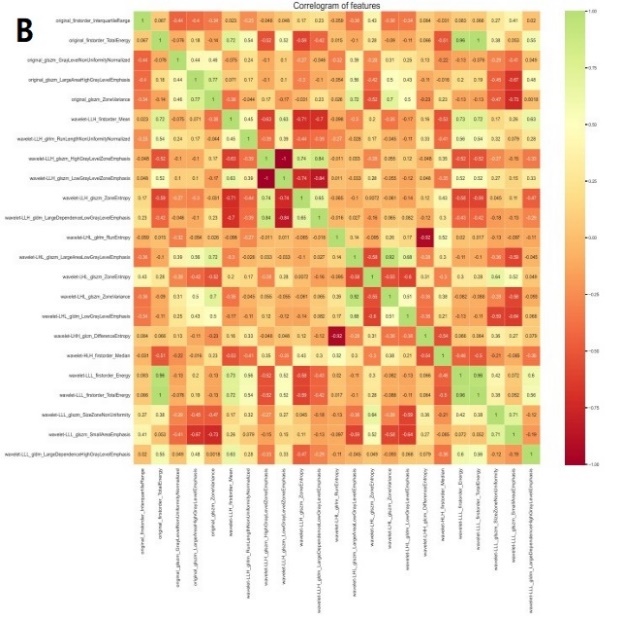


Figure S5. Screening results of myocardial texture characteristics in CA-LVH and HHD groups. (A) Weight coefficients of each myocardial texture feature. (B) Correlogram illustrating independence between each texture feature. Red squares represent negative correlations, while green squares represent positive correlations. The magnitude of the correlation between features is indicated by the color gradient.


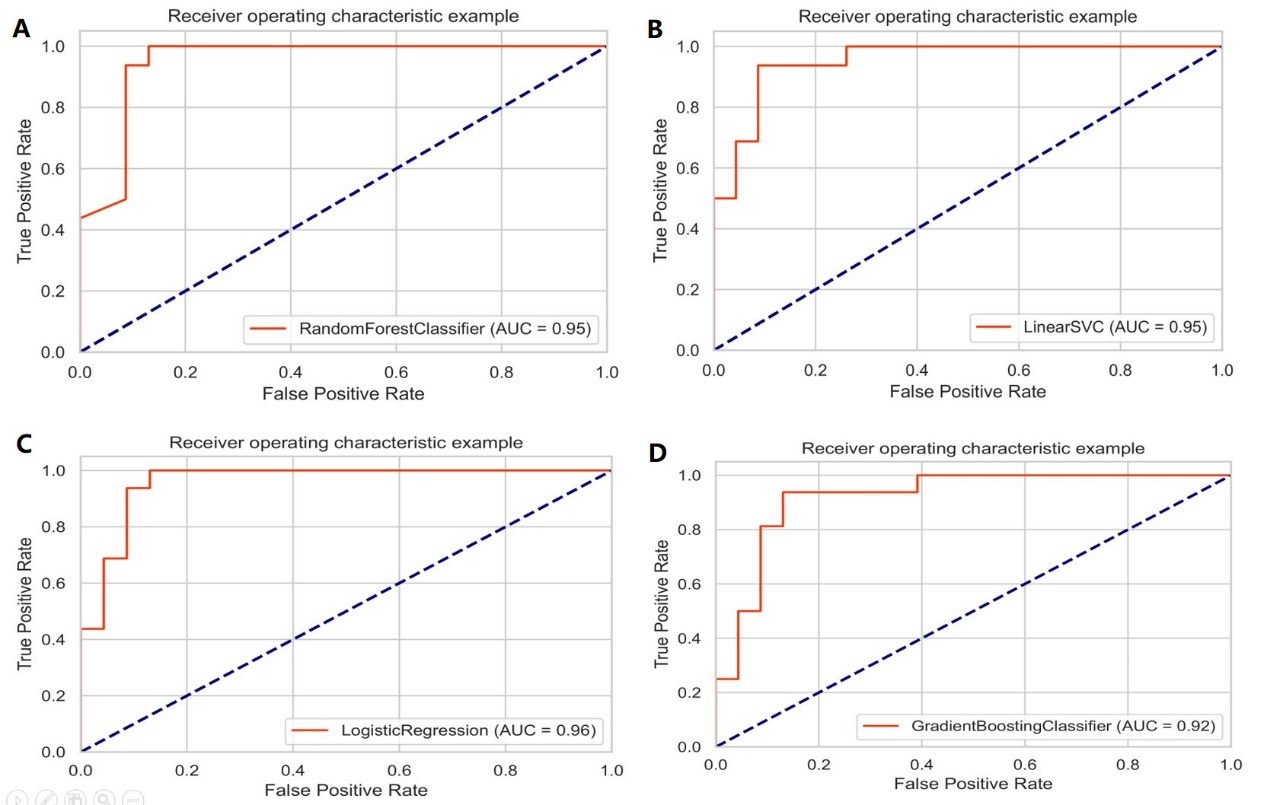


Figure S6. ROC curves of the ca-LVH group and HHD group models were constructed based on four algorithms. (A) Receiver operating characteristic curve of the prediction model based on random forest. (B) Receiver operation characteristic curve of the prediction model based on SVM. (C) Receiver operation characteristic curve based on logistic regression. (D) Receiver operation characteristic curve of the prediction model based on GBDT.
